# Supplementary material for: Patterns of Intron Gain and Loss in Fungi
Source: PLoS Biol. 2004 Nov 30;2(12):e422. doi: 10.1371/journal.pbio.0020422 (PMC532390; doi:10.1371/journal.pbio.0020422)
Supplement: Table S1 — Also available at http://genes.mit.edu/NielsenEtAl/. (4.3 MB ZIP). [file pbio.0020422.st001.zip › NielsenEtAl/html/84.html]

AN4018.1.NCU03108.1.MG03702.1.FG00925.1


```
 CLUSTAL W (1.82) Multiple Sequence Alignments - Introns Inserted


Sequence 1: MG03702.1	840 aa
Sequence 2: FG00925.1	787 aa
Sequence 3: NCU03108.1	810 aa
Sequence 4: AN4018.1	772 aa
Alignment Length: 889 aa
Number Identitical Residues: 316 aa
Alignment Score (without introns) 17486


MG03702.1 	MADITGK0VGK0HGKYINYITSEVSK0SRAGSMQQVDVACGKPRARSGSAEKAGPIYI0M
NCU03108.1	-------~---~--------------~-----------------------------MG~T
FG00925.1 	-------~---~--------------~-------------------------------~M
AN4018.1  	-------~---~--------------~------------------------MKEKAIM~S
          	                                                            

MG03702.1 	PSERTPLITTHQAGPQ-RQRYHHNVARRFCTIALSSTLIWVFFTWITTAILGRGP----A
NCU03108.1	PSEHTPLIATVEVGAAPRRRYSHHVARRFFTLLLSSILFWFFLFFIASFTFLP-------
FG00925.1 	PTESTPLIQTIRVGPP-RRRYPHQTWRRFFTLICSVVVIGGFGLFVIQTLLIG------P
AN4018.1  	PSETTPLLVPVQVAPQ-RHRYPHDKLRRACSYSLSLILAVALVLFLFPQALFPREGGSLW
          	*:* ***: . ....  *:** *.  **  :   *  :   :  ::    :    ..:  

MG03702.1 	HRHHGHGDWSWPGHKNRRLDDEGLRKVLTQTPSSELAEEWSRYYTSGPHLAGQNYSQ~AE
NCU03108.1	----RPSSWPGCGHGRSKVDYKHLRQLLLDTPSSKKAEEWQRYYTAGPHLAGKNYSQ0AL
FG00925.1 	HHHHGHPGSWLPGKS--HLSYDELERILFDTPDPKKAEEWSRYYTSGPHLAGKNLSQ0AE
AN4018.1  	SYLPGAQPYPNTWPSGNGLDQEELQTLLLGTPSAARAREWSKYYTSGPHLTGKNLSQ~AL
          	                  :. . *. :*  **..  *.**.:***:****:*:* ** * 

MG03702.1 	WTRNKWESWGIDS-SIVAYDVYLNRPVDHRLALLEKATGGKHDEDLSLSKPSWEVKFEAS
NCU03108.1	WTKEKWEEFGVQS-HIVDYEVYINYPVDHRLALLNKEDKKNDS-----SSDNWQVSFEAT
FG00925.1 	WTRDRWEEFGVAS-NIVAYDAYLNYPVDASVSILEKSKNGK----------GWNTTFKAS
AN4018.1  	WTKERWEEFGIADTKIATYDVYLNYPLDHRLALYQ----------------GGNISYEAS
          	**:::**.:*: .: *. *:.*:* *:*  ::: :                . : .::*:

MG03702.1 	LKEDIIDDDPTTALNNAIPTFHGYSASGNVTAQFVYVNYGTYQDFEDLVKANVSLKGKIA
NCU03108.1	LEEDILDEDPSTSLPDRVPVFHGYSASGNVTAPVVYVNYGTYQDFEDLLKANVSLAGKIA
FG00925.1 	LEEDIIEEDPTTSYDNRVPIFHGYSASGNVTASFVYVNYGTYQDYQDLVDAKVDVKGKIA
AN4018.1  	LEEDVLEEDSTSGLPDRVPTFHGYSASGNVTASFVFVNFGTYADFEDLVNANVSLSGKIA
          	*:**::::*.::.  : :* ************ .*:**:*** *::**:.*:*.: ****

MG03702.1 	IARYGGIFRGLKVKRAQELGMTATVIYSDPGDDGSTTDEKGEKQYPEGPARQASSVQRG-
NCU03108.1	IARYGLIFRGLKVKRAQELGMIGVIIFTDPGEDGEMTEENGYAPYPEGPARQPSSVQRGS
FG00925.1 	IARYGGIFRGLKIKRAQELGFVGVLIYSDPGDDGERTEEHGYKPYPEGPARNPSAVQRGS
AN4018.1  	IAKYGRVFRGLKVKRAQELGMVGVVLYDDPQTDGEYTEENGYKPYPEGPARNPSAVQRGS
          	**:** :*****:*******: ..::: **  **. *:*:*   *******:.*:****:

MG03702.1 	-----~--------~-------------------SAH2IPSIPISYADAIPILKALNGHG
NCU03108.1	TQFLS~V--APGDP2TTPGYPSKPGVPRGPVDGAIPS~IPSIPLSYAEALPILKALNGHG
FG00925.1 	AEFLS~I--RPGDP2STPGYPSKPDSPRVPVDDATAS~IPSIPISYRDALPILKALNGHG
AN4018.1  	TQFLS1IGFAPGDP~TTPGYPSKPGCERQDPHHFIPS~IPSIPVSNRDVLPLLKALNGHG
          	:.  :  .  .... ::.. .:...          .  *****:*  :.:*:********

MG03702.1 	PSSDEFNKYWTRNRGLQYKGVKYNVGPSPPGLVLNLYNEQNYITTPIWNVIGVINGTIED
NCU03108.1	PKASDFNKWWTARTGLGHKGVHYNIGPTPDDVVVNLYNEQEYVTTPLWNVIGIINGTIPD
FG00925.1 	PKSSDFNKYWNKNLGLKYKGIEYNIGPTPDDVVVNLYNEQKYVTTPIWDVIGIVNGTIPN
AN4018.1  	PKASDFNEAWQGG-GLAYKGVEYNIGPSPDDLVINLYNEQEYVTTPLWNVIGVIPGSLP-
          	*.:.:**: *    ** :**:.**:**:* .:*:******:*:***:*:***:: *::  

MG03702.1 	EVIV~VGNHRDAWIIGGAGDPNSGSAVINEVIRSFGKALESGWKPLRTIVFASWDGEEYS
NCU03108.1	EVIV1IGNHRDAWIAGGAGDPNSGSAVLNEVIRSFGEALRLGWKPMRTIVFASWDGEEYG
FG00925.1 	EVIV~VGNHRDAWIVGGAGDPNSGSAVLNEVIRGVGKAIDAGWKPLRTIVFASWDSEEYS
AN4018.1  	DTII~LGNHRDAWIAGGAGDPNSGSAVLNEVVRSFGEARRAGWKPLRTIVFASWDGEEYG
          	:.*: :******** ************:***:*..*:*   ****:*********.***.

MG03702.1 	LIGSTEWVEQYLPWLTNVNVAYINTDVAVAGPNFKASGAPLLDQLLYDITSQVQSPNQTV
NCU03108.1	LVGSTEWVEEYLPWLSAANVAYLNIDVGVRGKVFSASAAPLLNSIIYSATGAVESPDHPG
FG00925.1 	LIGSTEWVEEYLPWLSEANVAYVNVDVGVDGPEFTASAAPLLNQIIRDVTSAVPSPNQTV
AN4018.1  	LLGSTEWVEDHLPWLSKSNVAYLNVDVAASGTRLAPNASPLLNKLIYEITGLVQSPNQTV
          	*:*******::****:  ****:* **.. *  : ...:***:.:: . *. * **::. 

MG03702.1 	EGQTVRDVWDGRISTIGSGSDFTAFQDFAGIPCVDIGFSAHPDDAVYHYHSNYDSFYWMQ
NCU03108.1	H-SILEKNWDKKIDTMGSGSDFTAFQDFAGIPSLDVGFVAGEDDPVYHYHSNYDSFYWMK
FG00925.1 	PGQTVNDLWSGRITTMGSGSDFTAFQDFAGIPCIDFGFKYRSTSAVYHYHSNYDSFYWMK
AN4018.1  	PGQTVRDVWDGYIGTMGSGSDFTAFQDFAGIPSYDLGFSPSSQDPVYHYHSNYDSFDWMQ
          	 .. :.. *.  * *:****************. *.**     ..*********** **:

MG03702.1 	NFGDPGFVYHRTMAQILGLVVAKLADKPVIPFGVKNYADKLTSYIARVEDKLDAAMAG-I
NCU03108.1	KYGDPGFVYHRTMAQIFGLLIAELAHLPVLAFSAESYATALDGYVTQVEDKLNAVLHPPK
FG00925.1 	EYGDVGFKYHRTMAQILGLTIAKLAGTVIIPFGATEYADALNGYLDKVEAKFEPAT----
AN4018.1  	RFGDPDWLYHEACAKIWALAAAKLAETPVLFFNATDYSLGLEEYVDRIRPAADNLP----
          	.:** .: **.: *:* .*  *:**   :: *.. .*:  *  *: ::.   :       

MG03702.1 	TLESQPDLSTEQHLFELRASH---------HVDTTQPKGSAEGFKRELARLREAANTFSQ
NCU03108.1	EEEVDISTLTEQVISELRGNHYNSSSSSSTAITSSTSKATVKAFRDSFKRLHDAVDKLTE
FG00925.1 	------EVMSDDEIFSMRG-----------TVLSDKPIGSEHEFKKSLDHIRQQLGHFHL
AN4018.1  	-----------------------------------------NGLTFDFGPLYEAISRLQK
          	                                         . :  .:  : :  . :  

MG03702.1 	RAKAADHLANKLRKRVEDELPWWQWPAKIKLGWDIRKLNNKLKVIERQFLYSKGLDGRSW
NCU03108.1	KAARLDAEAEDLRSQAREHIPWYHWHQKIKLGYQIRKVNTKYKYIERSFLFSQGLDGRPW
FG00925.1 	KASELDAEAEIANHKLEEGLPWWNIIGKIRLGYTIERINKRYKFLERNFLYEGGLDGRDW
AN4018.1  	TAIEFDAYAADLTSQLTEELPWYLWWKKVRLFFLIHEVNTKYKNIERQFLYQQGLDGRSW
          	 *   *  *     :  : :**:    *::* : *..:*.: * :**.**:. ***** *

MG03702.1 	FK0SVIFAPGLWTGYAGA~VFPGLQESIDSKDFDNAI~RWVDIIEDCIYKAAGVLRLEDG
NCU03108.1	FK~HVVFAPGLWTGYSGA1VFPGLVESIDARDWSNAE~RWVDIIEACIKNAARKL-----
FG00925.1 	FK~HVVFAPGIWTGYSGA1VFPGWDESIDAKDYTNGL~KWSAIIVGCINKAIDSLSS---
AN4018.1  	FK~HVVFAPGLWTGYAGA~TYPGIVESLEAGDVANAA0KWQYIVIERVKAATELLQ----
          	**  *:****:****:** .:**  **::: *  *.  :*  *:   :  *   *     

MG03702.1 	E
NCU03108.1	-
FG00925.1 	-
AN4018.1  	-
          	
```
